# Grape Seed Extract as a Feed Additive Improves the Growth Performance, Ruminal Fermentation and Immunity of Weaned Beef Calves

**DOI:** 10.3390/ani13111876

**Published:** 2023-06-05

**Authors:** Jian Ma, Xue Fan, Wenjie Zhang, Guangxian Zhou, Fuquan Yin, Zhihui Zhao, Shangquan Gan

**Affiliations:** 1 College of Coastal Agricultural Sciences, Guangdong Ocean University, Zhanjiang 524088, China; crazyma0411@163.com (J.M.);; 2 College of Animal Science, Xinjiang Agricultural University, Urumchi 830052, China

**Keywords:** grape seed extract, weaned cross-breed beef calf, ruminal fermentation, antioxidant ability, immune response

## Abstract

**Simple Summary:**

Calf stage is critical to the production performance and meat quality of growing beef cattle. Early weaning is gradually carried out in rearing calves because it can accelerate their gastrointestinal development. Nevertheless, calves experience a significant stress reaction due to the transformation of feed type in weaning. Therefore, mitigating the weaning stress and promoting the healthy growth of weaned beef calves are of great significance for modern large-scale beef cattle farming. Our study evaluated the effects of grape seed extract supplementation on growth performance, ruminal fermentation, nutrient digestibility and serum biochemical, antioxidative and immune parameters of weaned cross-breed beef calves. The findings obtained from the current study indicate that grape seed extract as a feed additive led to a significant improvement in growth performance, ruminal fermentation, antioxidative capacity and immunity of weaned cross-breed beef calves.

**Abstract:**

The purpose of this research was to evaluate effects of grape seed extract (Gse) supplementation on the growth performance; ruminal fermentation; nutrient digestibility; and serum biochemical, antioxidative, and immune parameters of weaned beef calves. A total of 30 Simmental crossbred male calves with similar age and body weight were randomly allocated to two groups: a control group with no Gse (CON) and a Gse supplementation group (GSE) (4 g/d Gse per animal). The results show that, compared with the CON group, the average daily gain significantly increased (*p* = 0.043) in the GSE group. The ruminal contents of microbial protein and butyrate in GSE group were higher (*p* < 0.05) than those in the CON group. Additionally, calves fed Gse displayed increased (*p* < 0.05) dry matter and neutral detergent fiber digestibility. Moreover, the serum concentrations of triglyceride, catalase, superoxide dismutase, immunoglobulin G and immunoglobulin M were higher (*p* < 0.05) in the GSE group than those in the CON group. However, opposite tendencies of non-esterified fatty acid, malondialdehyde, tumor necrosis factor-α and interleukin-6 were found between the two groups. Overall, the supplementation of Gse can improve ruminal fermentation, nutrient digestibility, antioxidant ability, and immunity, as well as promoting the healthy growth of weaned cross-breed beef calves.

## 1. Introduction

Calf health is critical to the production performance and meat quality of growing cattle, which influences the sustainable development of the beef cattle industry. Early weaning is gradually conducted in rearing calves because it can accelerate the gastrointestinal development of calves and reduce production costs [1]. Nevertheless, calves experience a significant stress reaction due to the transformation of feed type after weaning. On the other hand, the gastrointestinal tract, particularly the rumen, of weaned calves is in the developmental process and vulnerable to changes in their living environment [2]. As a result, weaned calves have less resistance to harmful bacteria that can cause the dysfunction of digestive organs [3]. In addition, the immune system of weaned calves is not fully developed; therefore, calves cannot effectively regulate their immunity to maintain healthy growth. Previously, a study on dairy calves found that weaning could cause an inflammatory reaction and increase concentrations of inflammatory factors, including interleukin-1β (IL-1β), IL-6, and tumor necrosis factor-α (TNF-α) in blood [4]. These adverse influences caused by weaning can ultimately affect the healthy growth of calves and decrease the productivity of growing cattle. Therefore, mitigating the weaning stress and promoting the healthy growth of weaned beef calves are of great significance for beef cattle farming.

Grape seed extract (Gse) is a kind of polyphenol extracted and isolated from grape seeds, mainly including procyanidin, catechin, gallic acid and epicatechin. Recently, the utilization of Gse has become widespread because of its various antimicrobial, anti-inflammatory and antioxidative abilities [5]. In dairy cows, the dietary supplementation of 1% grape seed and grape marc meal extract (dry matter basis) had no significant effects on dry matter intake (DMI) but increased the milk production [6]. In lambs, the addition of grape seed procyanidin in the ration at 20 mg/kg body weight (BW) can increase DMI (1.89 kg/d vs. 1.99 kg/d) and average daily gain (ADG) (280.4 g/d vs. 315.9 g/d) [7]. Due to being rich in polyphenols, a previous study reported that Gse supplementation could enhance the antioxidant ability and feed efficiency of rabbits under heat stress [8]. Furthermore, Gse was verified to regulate inflammatory responses by decreasing the gene expressions of pro-inflammatory cytokines in the intestine [9]. A recent study found that dietary supplementation of Gse could improve energy metabolism and colonic fermentation, thus promoting the healthy growth of pre-weaning dairy calves under heat stress [10]. Nevertheless, the influences of Gse supplementation on the ruminal fermentation, nutrient digestibility and immunity of weaned calves have not been fully investigated.

In calves, the rumen, which plays an important role in the digestion and absorption of volatile fatty acid (VFA) and ammonia nitrogen (NH_3_-N), is immature. The ruminal development is strongly linked to VFA yield and absorption, which is main energy source for ruminants [11]. An in vitro study demonstrated that Gse could regulate VFA production and affect ruminal fermentation [12]. As mentioned earlier, weaning can trigger oxidative stress and inflammatory responses and down-regulate the immunity of calves. Previous experiments in cows, calves and lambs reported that the supplementation of grape seed products could reduce inflammatory reactions and improve oxidation resistance, and thus is conducive to promoting production performance [10,13,14]. Although Gse can improve the production performance and antioxidant ability of animals, research on the role of Gse as an effective feed additive in young ruminants is limited. According to previous experiments, we hypothesized that Gse may be a promising feed additive than can promote the healthy growth of weaned cross-breed beef calves. Thus, the present research was carried out to explore the influence of Gse supplementation on growth performance, ruminal fermentation, and nutrient digestibility, as well as the biochemical, antioxidative and immune parameters of serum, in weaned cross-breed beef calves.

## 2. Materials and Methods

### 2.1. Experimental Animals and Diet and Feeding Management

In the current study, a total of 30 healthy Simmental cross-breed (Simmental ♂ × Qinchuan cattle ♀) calves of a similar age (68 ± 6 d) and BW (83.47 ± 4.59 kg) after weaning were used. All animals were uncastrated male calves. After marking with ear tags, the calves were randomly divided into 2 groups: control with no Gse (CON) and Gse treatment group (GSE) (4 g/d Gse (Beisong Plant Technology Co., Ltd., Xi’an, Shanxi, China; main ingredients: procyanidin 50%; catechin 24%; gallic acid 16%; epicatechin 6%) per animal). The additive amount of Gse was based on the previous study in pre-weaning dairy calves [10].

All animals subject to 2 treatments were reared in 30 pens with 1 calf in each pen. Calves were fed with total mixed ration three times daily at 07:30, 13:30 and 19:30, respectively, and had unlimited access to water. The feeding trial was conducted with a 10 d adaptive phase followed by 60 days of experimental period. The Gse was mixed with the basal diet, which was designed based on the NRC [15]. The feed ingredients and nutritional levels of the experimental diet are shown in Table 1 and Table 2.

### 2.2. Body Weight Measurement

Before morning feeding, the BW of beef calves was determined on d 0 and 60, and the ADG was obtained according to initial and final BW. During the experiment, the feed intake of each calf was recorded according to the difference in feed both offered and refused, and then converted into DMI. Feed efficiency was calculated via dividing DMI by ADG.

### 2.3. Serum, Ruminal Fluid and Fecal Samples Collection

On d 0 and 60, blood samples of each animal were obtained from the jugular vein before morning feeding. Subsequently, blood samples were centrifuged (3000 rpm and 4 °C for 15 min) to collect serum. The serum samples were preserved in 1.5 mL centrifuge tubes (−20 °C). On d 60, the ruminal fluid of each calf was sampled at 4 h after morning feeding. The initial 100 mL fluids collected using an esophageal tube were thrown away [16]. The pH of fluid was determined using a pH meter (Ruizhen Electronic Technology Co., Ltd., Shanghai, China). Next, the fluid samples were filtered with 4 layers of cheesecloth, preserved in 10 mL centrifuge tubes, and stored at −20 °C for the measurement of ruminal fermentation characteristics.

Fecal samples of all calves were collected from d 57 to 59 according to the method described in our previous study [1]. Briefly, feces were sampled at 6 h intervals from rectums of calves and the sampling time are as follows: d 57, 21:00, 03:00, 09:00 and 15:00; d 58, 19:00, 01:00, 07:00 and 13:00; and d 59, 17:00, 23:00, 05:00 and 11:00. During the collection of feces, fresh diets and orts were collected. The daily diets, orts and feces from each animal were blended, subsampled, and stored at −20 °C. Lastly, all samples (the 100 g feces were blended with 10 mL of 10% sulphuric acid) were dried at 65 °C in a forced-air oven for 48 h to a constant weight [17]. Subsequently, dried samples were smashed in order to pass them through a 1 mm sieve (Taifeng Machinery Equipment Co., Ltd., Yantai, China) for analysis of nutrient digestibility.

### 2.4. Serum and Ruminal Fluid Samples Analysis

The concentrations of total protein (TP), albumin (ALB), alanine transaminase (ALT), aspartate transaminase (AST), alkaline phosphatase (ALP), glucose (GLU), triglyceride (TG), non-esterified fatty acid (NEFA) and urea nitrogen (UN), in serum samples were determined using an automatic biochemical analyzer (BS350, Baden Medical Co., Ltd., Nanjing, China). In addition, the contents of glutathione peroxidase (GSH-Px), superoxide dismutase (SOD), catalase (CAT), malondialdehyde (MDA), total antioxidant capacity (T-AOC), immunoglobulin A (IgA), IgG, IgM, IL-1β, IL-6, IL-10 and TNF-α were measured using commercial kits (Nanjing Jiancheng Bioengineering Institute, Nanjing, China) according to the instructions. After unfreezing, the ruminal fluids were centrifuged (15,000 rpm and 4 °C for 12 min) to obtain the supernatant for determining VFA [18], microbial protein (MCP) [19] and NH_3_-N [20] contents.

### 2.5. Nutrient Digestibility Analysis

The dry matter (DM, method 934.01), organic acid (OM, method 942.05), crude protein (CP, method 984.13) and ether extract (EE, method 954.02) contents in diets, orts and feces were measured based on the AOAC procedure [21]. Additionally, the concentrations of neutral detergent fiber (NDF) and acid detergent fiber (ADF) were analyzed using the methods of Van Soest et al. [22]. Moreover, the acid-insoluble ash (AIA) content in diet and feces was measured using the procedure of Van Keulen and Young [23]. The AIA ratio technique was used to determine nutrient digestibility, and our calculation method is described in our previous study [1].

### 2.6. Statistical Analysis

Firstly, normality and homogeneity of variance tests were carried out on all data. Subsequently, the data for growth performance, ruminal fermentation, nutrient digestibility, and serum parameters were analyzed using an independent sample *t*-test via SPSS software. The results are presented as means and standard error of means. Statistical differences were considered significant for *p* < 0.05 and as a tendency for 0.05 ≤ *p* < 0.10.

## 3. Results

### 3.1. Growth Performance

As shown in Table 3, the initial and final BWs of the two groups were similar (*p* > 0.05). Compared with the CON group, the ADG of the GSE group significantly increased (*p* = 0.043). No significant difference (*p* > 0.05) of DMI was found between the CON and GSE groups, while the ratio of DMI to ADG in the GSE group was slightly lower (*p* = 0.063) than that in the CON group.

### 3.2. Ruminal Fermentation

**Figure 1:**
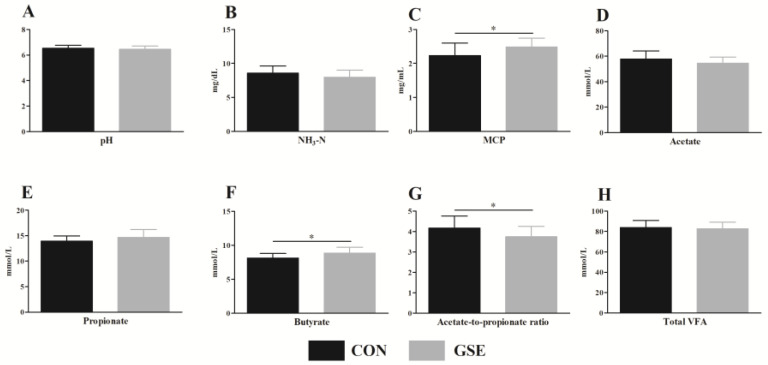
Effects of grape seed extract on ruminal fermentation of weaned beef calves. (**A**) pH; (**B**) NH_3_-N, ammonia nitrogen; (**C**) MCP, microbial protein; (**D**) acetate; (**E**) propionate; (**F**) butyrate; (**G**) acetate-to-propionate ratio; (**H**) total VFA, volatile fatty acid. Gse, grape seed extract. CON, control with no Gse (Beisong Plant Technology Co., Ltd., Xi’an, Shanxi, China) and fed basal diet; GSE, fed basal diet and 4 g/d Gse per calf. The asterisk (*) indicates a significant difference between the CON and GSE groups (*p* < 0.05).

Ruminal pH was similar (*p* > 0.05) and averaged 6.53 and 6.45 in the CON and GSE groups, respectively (Figure 1A). Likewise, there were no significant differences (*p* > 0.05) in NH_3_-N (Figure 1B), acetate (Figure 1D), propionate (Figure 1E) and total VFA (Figure 1H) concentrations between the CON and GSE treatments. Nevertheless, the ruminal MCP (Figure 1C) and butyrate (Figure 1F) contents were higher (*p* < 0.05) than those of the CON group. Conversely, the acetate-to-propionate ratio (Figure 1G) in the CON group increased by 11.2% (*p* < 0.05) when compared to the GSE group.

### 3.3. Nutrient Digestibility

**Figure 2:**
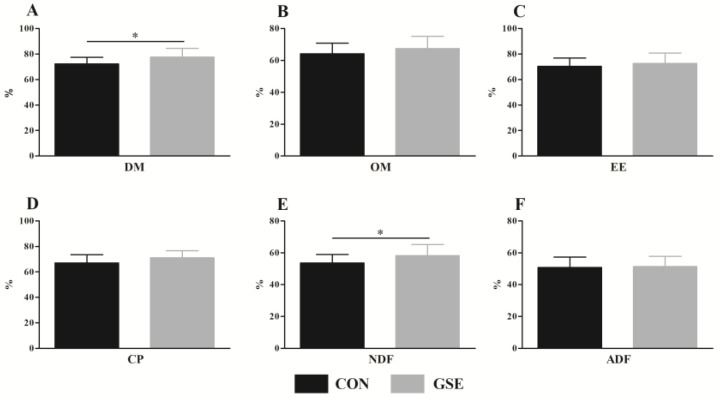
Effects of grape seed extract on nutrient digestibility of weaned beef calves. (**A**) DM, dry matter; (**B**) OM, organic matter; (**C**) EE, ether extract; (**D**) CP, crude protein; (**E**) NDF, neutral detergent fiber; (**F**) ADF, acid detergent fiber. Gse, grape seed extract. CON, control with no Gse (Beisong Plant Technology Co., Ltd., Xi’an, Shanxi, China) and fed basal diet; GSE, fed basal diet and 4 g/d Gse per calf. The asterisk (*) indicates a significant difference between the CON and GSE groups (*p* < 0.05).

The apparent digestibility of OM (Figure 2B), EE (Figure 2C) and ADF (Figure 2F) were not different (*p* > 0.05) in the CON and GSE groups. However, Gse supplementation significantly increased (*p* < 0.05) DM (Figure 2A) and NDF (Figure 2E) digestibility. Additionally, CP (Figure 2D) digestibility in the GSE group tended to be higher (*p* = 0.077) than that in the CON group.

### 3.4. Serum Biochemical Parameter

Evidently, on d 0, no significant differences (*p* > 0.05) in all serum biochemical parameters were found between the CON and GSE treatments (Table 4). On d 60, calves fed Gse had significantly increased (*p* = 0.021) serum TG content. However, the NEFA content of GSE group was lower (*p* = 0.018) than CON group. In addition, calves fed Gse tended to have a higher (*p* = 0.091) serum GLU content.

### 3.5. Serum Antioxidant Parameter

On d 0, there were no significant differences (*p* > 0.05) in GSH-Px, CAT, SOD, MDA and T-AOC concentrations between CON and GSE groups (Table 5). On d 60, Gse supplementation significantly enhanced (*p* < 0.05) the concentrations of CAT, SOD and T-AOC in serum of calves. Nevertheless, the MDA activity displayed an opposite tendency between two groups. Moreover, the GSH-Px activity of GSE group was slightly higher (*p* = 0.078) than that of CON group.

### 3.6. Serum Immunoglobulin

The concentrations of serum immunoglobulin were similar (*p* > 0.05) between CON and GSE treatments on d 0 (Table 6). Nevertheless, the IgG and IgM contents of GSE group were higher (*p* < 0.05) than those of CON group on d 60. Furthermore, compared with CON group, a slight elevation (*p* = 0.074) of IgA content was observed in GSE group.

### 3.7. Serum Cytokine

Table 7 shows the effects of Gse supplementation on serum cytokine concentrations of weaned beef calves. On d 0, the TNF-α, IL-1β, IL-6 and IL-10 concentrations were similar (*p* > 0.05) in serum between two groups. On d 60, the TNF-α and IL-6 concentrations of GSE treatment were lower (*p* < 0.05) than those of CON group. Moreover, calves fed Gse had slightly reduced (*p* = 0.055) IL-1β content and increased (*p* = 0.054) IL-10 content.

## 4. Discussion

In calves, the digestive and immune functions are in developmental phases, and they are easily affected by external environments or pathogens. After weaning, calves undergo greatly physiological challenges, resulting in stunted growth, decreased immunity and higher morbidity [24]. Relieving the weaning stress of weaned calves is of great significance for cattle farming. As a natural antioxidant, Gse has various health benefits, including free radical scavenging activity, antimicrobial and anti-inflammatory activities [5]. A previous study reported that the dietary supplementation of grape seed procyanidin could increase the final BW, DMI and ADG in finishing lambs [7]. In our study, Gse supplementation increased the ADG of weaned cross-breed beef calves, while it had no significant difference on final BW and DMI. The reason that different results may be found is because the growth stages (young stage vs. fattening stage) of animals are different. On the other hand, the findings of the present study show that feed efficiency was slightly ameliorated by Gse supplementation. In a previous study, the addition of Gse improved the feed efficiency of calves suffering from heat stress [25], which is in accordance with our study. The polyphenols in Gse can inhibit the growth of pathogenic bacteria (e.g., *Escherichia coli* and *Salmonella*) in the digestive tract of animals [26], which is helpful for decreasing the incidence rate of diarrhea. The better growth performance of ruminants is commonly related to improved digestive ability and immunity. Thus, the following experiments were carried out to explore the effects of Gse supplementation on ruminal fermentation, nutrient digestibility, antioxidant ability and immunity of weaned beef calves.

The ruminal pH, which affects the growth and proliferation of microorganisms and regulates VFA production, can be used to assess the healthy status of rumen within a normal range 6 to 7 [27]. Previously, a study found that supplementing low forage diets with grape marc could decrease ruminal pH [28]. Nevertheless, in our study, the ruminal pH values of the CON and GSE groups were within the normal range from 6.45 to 6.53, indicating that Gse supplementation had no negative effects on ruminal fermentation. The difference in research results may be associated with the dietary ratio of concentrate to roughage. Furthermore, calves fed Gse increased MCP content in the rumen. Plant polyphenols can reduce the degradation of protein in the rumen and increase intestinally absorbable dietary protein [29], which can explain the improvement in ruminal MCP. In the rumen, NH_3_-N is the main raw material that synthesizes MCP. An in vitro study reported that polyphenols can reduce the ruminal NH_3_-N content [30]. In our research, the NH_3_-N concentration was similar between the two treatments. This suggests that the microbial communities in calves were still in developmental period.

In the present study, Gse supplementation significantly increased the ruminal butyrate content of weaned cross-breed beef calves. Butyrate is the main energy source of the ruminal epithelium, and ruminal development is closely related to the butyrate content produced by microorganisms [31]. Therefore, the elevated content of butyrate found in this study might be helpful for the ruminal development of weaned cross-breed beef calves. In addition, we found that the ratio of acetate to propionate in the GSE group was reduced compared to the CON group, indicating that Gse supplementation promoted the ruminal propionate fermentation of calves. In ruminants, propionate is an important precursor of gluconeogenesis [32]. Ruminal propionate fermentation can provide more energy for the body. Moreover, a previous study reported that grape seed meal could up-regulate the relative abundance of butyrate-producing bacteria, which contributed to the increased content of butyrate in the rumen [33]. In the future, the effects of Gse on microbial communities in the rumen of calves require an in-depth investigation.

In addition to rumen fermentation, nutrient digestibility could also directly affect the growth performance of calves. DM and OM digestibility are important indexes that can be used to measure animal diets [34]. In our study, Gse supplementation significantly increased DM digestibility, indicating that the calves in the GSE group could obtain more nutrients and promote growth, which matched with growth performance data. Moreover, the NDF digestibility of calves fed with Gse significantly increased, which was in line with the study by Juráček et al. [35], who reported that the dietary supplementation of grape pomace could improve the NDF digestibility of wethers. In dairy calves, the supplementation of gallic acid, an ingredient of Gse, in the starter feed of calves, is confirmed to up-regulate the relative abundance of fecal *Ruminococcaceae*, *Bacteroides* and *Christensenellaceae* [36], which have essential functions in cellulose degradation and VFA production. We speculated that the positive effects of grape seed products on NDF digestibility might be related to the regulation of microbiota in the digestive tract. Forage can be utilized by ruminal microorganisms to generate MCP and small peptides that are easy to absorb by small intestine to increase nutrient digestibility [37]. Interestingly, Gse supplementation slightly increased the CP digestibility, which was consistent with MCP result mentioned earlier. Generally, improved nutrient digestibility improves ADG, as seen in growth performance results. Lastly, the activity of digestive enzymes plays a vital role in nutrient digestion. Thus, future research should be carried out to elucidate the effects of Gse supplementation on the digestive enzyme activity of calves.

As important parameters associated with the health of animals, serum biochemistry reflects the physiological metabolism of the body and changes in various organ functions [38]. The serum contents of TP, ALB, GLB, and UN are essential indexes of protein metabolism, and changes in GLU, TG, and NEFA concentrations in serum are closely related to lipid and energy metabolism. Moreover, the ALP, ALT and AST concentrations in serum reflect hepatic functions [38]. In this study, the serum concentrations of TP, ALB, GLB, UN, ALP, ALT, and AST were similar between two groups, suggesting that Gse supplementation did not have negative effects on protein metabolism and the hepatic function of weaned beef calves. In laboratory animals, one study showed that Gse could protect the liver when its function was experimentally damaged [39]. In ruminants, Nudda et al. [40] found that dietary supplementation with grape seed did not have significant effects on UN, ALT, and AST concentrations in blood of dairy ewes, in accordance with our results. Our study also found that Gse supplementation increased serum GLU and TG contents, while decreasing NEFA content in calves, indicating that Gse could improve energy metabolism. The reason for this may be related to the ruminal propionate fermentation caused by Gse supplementation, and ruminal propionate fermentation enhances the gluconeogenesis process, which can result in an elevated GLU content in the sera of calves. However, the potential mechanism of action still needs investigation. TG is the the most abundant and productive energy substance in the body, and under stress, the body will decompose TG into NEFA to supply energy for physiological activities via blood circulation [41]. Gse supplementation improved the negative energy balance of calves induced by weaning, which was beneficial for promoting growth.

Weaning calves are exposed to oxidative stress [42]. The GSH-Px, CAT, SOD, MDA, and T-AOC are the most important indexes used to determine the antioxidant ability of animals to protect themselves against oxidative damage [1]. In this experiment, Gse supplementation increased the serum activity of GSH-Px, CAT, SOD, and T-AOC and reduced MDA concentration, suggesting that Gse could improve the antioxidant ability of weaned cross-breed beef calves. In calves and lambs, previous studies reported that dietary supplementation with grape seed products effectively increased he serum antioxidative enzymes [25,43], which is consistent with our results. Plant polyphenols have been demonstrated to protect animals from oxidative damage via the Nrf2/Keap1 signaling pathway [44]. In addition, catechin, gallic acid and procyanidins, which are the main phenolic ingredients of Gse, inhibit the production of reactive oxygen species and free radicals mainly by scavenging hydrogen peroxide and free radicals and preventing macromolecular damage [5]. A recent study found that lambs supplemented with grape seed procyanidin had an enhanced activity of CAT, SOD, and T-AOC and reduced MDA content in muscle [7], which is essentially in line with the results of our study. Our results show that Gse supplementation has positive effects on CAT, SOD, MDA, and T-AOC in calves under weaning stress.

Due to undeveloped digestive and immune functions, calves usually have reduced immunity [24]. Serum immunoglobulin content reflects the immune function of animals. At present, research on the effects of Gse on the immunity of ruminants remains scare. In our study for heightening the immunologic function of calves. The polyphenols of Gse can inhi, calves fed Gse had increased serum immunoglobulin (IgA, IgG and IgM) contents. Serum immunoglobulins have the key function of humoral immunity. Therefore, elevated immunoglobulin contents were beneficial for the growth of harmful microorganisms, including *Escherichia coli* and *Salmonella*, in the digestive tract of animals [26], which might explain the positive influence of Gse on immunity.

After weaning, due to the changes in feeding methods and feedstuffs, the integrity of tight junction protein that forms the gastrointestinal epithelium in animals is damaged. As a result, lipopolysaccharides enter into the blood circulation because of elevated gastrointestinal permeability, cause the release of pro-inflammatory cytokines, and trigger local and systemic inflammation [45]. As key pro-inflammatory factors, TNF-α, IL-1β, and IL-6 regulate inflammatory reactions of the body. IL-10 can inhibit the production of pro-inflammatory factors and the combination of factors with receptors, thus decreasing the damage of inflammatory reactions in the body [46]. In the present study, the dietary supplementation with Gse reduced the contents of TNF-α and IL-6 and slightly increased the IL-10 content in serum of calves, indicating that Gse alleviated the inflammatory responses of beef calves. In preweaning dairy calves, Gse is verified to reduce the blood concentration of TNF-α and attenuate the inflammatory response under heat stress condition [25], which were consistent with our findings. Another study found that grape seed product could decrease the level of pro-inflammatory cytokines by suppressing the NF-κB signaling pathway [47]. The beneficial influence of Gse on the regulation of inflammatory factors may be achieved by inhibiting the NF-κB signaling pathway. Future research should pay more attention to the potential mechanisms of Gse in the immunity of calves.

## 5. Conclusions

The results from the current study show that Gse supplementation improved the ADG, feed efficiency and DM and NDF digestibility of weaned beef calves. Moreover, calves fed Gse had higher ruminal MCP and butyrate contents. The antioxidant activity and immune responses of weaned beef calves were enhanced, as shown via the reductions in TNF-α, IL-6, and MDA concentrations and increases in CAT, SOD, IgG, and IgM concentrations in serum. Based on our study, Gse is an effective feed additive for improving the healthy growth of weaned beef calves.

## Figures and Tables

**Table 1:** Feed ingredients of experimental diet (DM basis, %).

Ingredients	
Alfalfa hay	48.00
Wheat straw	17.00
Corn	14.90
Wheat bran	6.30
Soybean meal	5.25
Cottonseed meal	3.55
Distillers dried grains with soluble	3.12
NaCl	0.18
Premix ^1^	1.70

^1^ The premix provided following per kilogram of diet: Fe 100 mg, Zn 80 mg, Mn 30 mg, Cu 10 mg, I 0.60 mg, Se 0.30 mg, Co 0.10 mg, VA 8000 IU, VD 1500 IU, VE 50 IU.

**Table 2:** Nutritional levels of experimental diet (DM basis).

Nutrient Levels	
Neg ^1^, MJ/kg	4.46
CP (%)	14.19
NDF (%)	42.85
ADF (%)	31.54
EE (%)	2.26
Ca (%)	0.75
P (%)	0.40

DM, dry matter; CP, crude protein; EE, ether extract; NDF, neutral detergent fiber; ADF, acid detergent fiber; NEg, net energy for gain. ^1^ NEg was a calculated value; the other nutrient levels were measured values.

**Table 3:** Effects of grape seed extract on growth performance of weaned beef calves.

Items	Treatments		SEM	*p*-Value
	CON	GSE		
Initial BW (kg)	91.93	92.27	0.974	0.868
Final BW (kg)	157.47	162.20	1.730	0.176
ADG (kg/d)	1.09	1.17	0.018	0.043
DMI (kg/d)	5.94	6.07	0.055	0.263
FE	5.47	5.23	0.065	0.063

BW, body weight; ADG, average daily gain; DMI, dry matter intake; SEM, standard error of mean; Gse, grape seed extract. CON, control with no Gse (Beisong Plant Technology Co., Ltd., Xi’an, Shanxi, China) and fed basal diet; GSE, fed basal diet and 4 g/d Gse per calf. FE = DMI/ADG.

**Table 4:** Effects of grape seed extract on serum biochemical parameters of weaned beef calves.

Items	Treatments		SEM	*p*-Value
	CON	GSE		
Day 0				
TP (g/L)	57.47	56.92	0.792	0.738
ALB (g/L)	30.05	30.80	0.645	0.567
GLB (g/L)	27.42	26.12	0.759	0.401
GLU (mmol/L)	3.99	4.06	0.054	0.507
TG (mmol/L)	0.297	0.282	0.005	0.193
NEFA (mmol/L)	0.197	0.189	0.010	0.734
UN (mmol/L)	3.41	3.28	0.067	0.344
ALP (U/L)	52.40	52.16	0.819	0.885
ALT (U/L)	12.44	12.92	0.203	0.247
AST (U/L)	42.21	43.14	0.705	0.518
Day 60				
TP (g/L)	58.79	59.73	0.795	0.563
ALB (g/L)	29.14	28.45	0.761	0.657
GLB (g/L)	29.65	31.29	0.636	0.205
GLU (mmol/L)	4.09	4.25	0.047	0.091
TG (mmol/L)	0.26	0.29	0.006	0.021
NEFA (mmol/L)	0.18	0.15	0.008	0.018
UN (mmol/L)	3.39	3.40	0.061	0.899
ALP (U/L)	51.57	52.18	0.846	0.725
ALT (U/L)	12.23	12.42	0.241	0.709
AST (U/L)	43.57	43.09	0.647	0.720

TP, total protein; ALB, albumin; GLB, globulin; GLU, glucose; TG, triglyceride; NEFA, non-esterified fatty acid; UN, urea nitrogen; ALP, alkaline phosphatase; ALT, alanine transaminase; AST, aspartate transaminase; SEM, standard error of mean; Gse, grape seed extract. CON, control with no Gse (Beisong Plant Technology Co., Ltd., Xi’an, Shanxi, China) and fed basal diet; GSE, fed basal diet and 4 g/d Gse per calf.

**Table 5:** Effects of grape seed extract on serum antioxidant parameters of weaned beef calves.

Items	Treatments		SEM	*p*-Value
	CON	GSE		
Day 0				
GSH-Px (U/mL)	167.32	169.82	3.642	0.738
CAT (U/mL)	15.55	16.42	0.658	0.519
SOD (U/mL)	77.83	74.44	1.262	0.183
MDA (nmol/mL)	6.26	6.28	0.134	0.936
T-AOC (U/mL)	5.55	5.29	0.132	0.338
Day 60				
GSH-Px (U/mL)	162.38	175.11	3.617	0.078
CAT (U/mL)	17.82	20.40	0.554	0.017
SOD (U/mL)	73.17	78.01	0.973	0.010
MDA (nmol/mL)	5.37	4.59	0.179	0.027
T-AOC (U/mL)	5.79	6.46	0.170	0.047

GSH-Px, glutathione peroxidase; CAT, catalase; SOD, superoxide dismutase; MDA, malondialdehyde; T-AOC, total antioxidant capacity; SEM, standard error of mean; Gse, grape seed extract. CON, control with no Gse (Beisong Plant Technology Co., Ltd., Xi’an, Shanxi, China) and fed basal diet; GSE, fed basal diet and 4 g/d Gse per calf.

**Table 6:** Effects of grape seed extract on serum immunoglobulin contents of weaned beef calves (μg/mL).

Items	Treatments		SEM	*p*-Value
	CON	GSE		
Day 0				
IgA	292.89	290.69	4.868	0.826
IgG	478.58	475.36	5.817	0.787
IgM	16.41	17.06	0.233	0.170
Day 60				
IgA	288.68	304.78	4.513	0.074
IgG	485.65	517.43	6.045	0.006
IgM	17.76	19.05	0.284	0.021

IgA, immunoglobulin A; IgG, immunoglobulin G; IgM, immunoglobulin M; SEM, standard error of mean; Gse, grape seed extract. CON, control with no Gse (Beisong Plant Technology Co., Ltd., Xi’an, Shanxi, China) and fed basal diet; GSE, fed basal diet and 4 g/d Gse per calf.

**Table 7:** Effects of grape seed extract on serum cytokine contents of weaned beef calves (pg/mL).

Items	Treatments		SEM	*p*-Value
	CON	GSE		
Day 0				
TNF-α	428.73	440.15	6.206	0.367
IL-1β	73.96	71.46	1.044	0.237
IL-6	235.76	223.63	4.052	0.137
IL-10	115.06	118.69	2.640	0.501
Day 60				
TNF-α	439.05	413.73	6.031	0.033
IL-1β	69.53	64.94	1.203	0.055
IL-6	224.80	213.29	2.809	0.038
IL-10	111.51	122.38	2.838	0.054

TNF-α, tumor necrosis factor-α; IL-1β, interleukin-1β; IL-6, interleukin-6; IL-10, interleukin-10; SEM, standard error of mean; Gse, grape seed extract. CON, control with no Gse (Beisong Plant Technology Co., Ltd., Xi’an, Shanxi, China) and fed basal diet; GSE, fed basal diet and 4 g/d Gse per calf.

## Data Availability

The data are available from the corresponding author.
